# Cardiovascular fingolimod effects on rapid baroreceptor unloading are counterbalanced by baroreflex resetting

**DOI:** 10.1007/s10072-020-05004-1

**Published:** 2021-01-14

**Authors:** Max J. Hilz, Sankanika Roy, Carmen de Rojas Leal, Mao Liu, Francesca Canavese, Klemens Winder, Katharina M. Hoesl, De-Hyung Lee, Ralf A. Linker, Ruihao Wang

**Affiliations:** 1 Department of Neurology, University of Erlangen-Nuremberg, Schwabachanlage 6, D-91054 Erlangen, Germany; 2 Department of Neurology, Icahn School of Medicine at Mount Sinai, New York, NY USA; 3 Department of Internal Medicine, Nottingham University Hospitals, Nottingham, UK; 4 Department of Neurology, Hospital Universitario Virgen de la Victoria, University of Malaga, Malaga, Spain; 5 Department of Neurology, Tongji Hospital, Tongji Medical College, HUST, Wuhan, People’s Republic of China; 6 Department of Psychiatry and Psychotherapy, Paracelsus Medical University, Nuremberg, Germany; 7 Department of Neurology, University Hospital Regensburg, University of Regensburg, Regensburg, Germany

**Keywords:** Cardiovascular fingolimod effects, Multiple sclerosis, Baroreflex gain, Valsalva maneuver, Baroreflex resetting

## Abstract

**Background and purpose:**

Initial cardiovascular fingolimod effects might compromise baroreflex responses to rapid blood pressure (BP) changes during common Valsalva-like maneuvers. This study evaluated cardiovascular responses to Valsalva maneuver (VM)-induced baroreceptor unloading and loading upon fingolimod initiation.

**Patients and methods:**

Twenty-one patients with relapsing-remitting multiple sclerosis performed VMs before and 0.5, 1, 2, 3, 4, 5, and 6 hours after fingolimod initiation. We recorded heart rate (HR) as RR intervals (RRI), systolic and diastolic BP (BPsys, BPdia) during VM phase 1, VM phase 2 early, VM phase 2 late, and VM phase 4. Using linear regression analysis between decreasing BPsys and RRI values during VM phase 2 early, we determined baroreflex gain (BRG) reflecting vagal withdrawal and sympathetic activation upon baroreceptor unloading. To assess cardiovagal activation upon baroreceptor loading, we calculated Valsalva ratios (VR) between maximal and minimal RRIs after strain release. Analysis of variance or Friedman tests with post hoc analysis compared corresponding parameters at the eight time points (significance: *p* < 0.05).

**Results:**

RRIs at VM phase 1, VM phase 2 early, and VM phase 2 late were higher after than before fingolimod initiation, and maximal after 4 hours. Fingolimod did not affect the longest RRIs upon strain release, but after 3, 5, and 6 hours lowered the highest BPsys values during overshoot and all BPdia values, and thus reduced VRs. BRG was slightly higher after 3 and 5 hours, and significantly higher after 4 hours than before fingolimod initiation.

**Conclusions:**

VR-decreases 3–6 hours after fingolimod initiation are physiologic results of fingolimod-associated attenuations of BP and HR increases at the end of strain and do not suggest impaired cardiovagal activation upon baroreceptor loading. Stable and at the time of HR nadir significantly increased BRGs indicate improved responses to baroreceptor unloading. Thus, cardiovascular fingolimod effects do not impair autonomic responses to sudden baroreceptor loading or unloading but seem to be mitigated by baroreflex resetting.

## Introduction

Fingolimod, a sphingosine-1-phosphate (S1P) receptor modulator, approved for treating patients with relapsing-remitting multiple sclerosis (RRMS) [21], has an initially vagomimetic effect on the heart [13, 22, 32, 33]. In healthy individuals as well as RRMS patients, fingolimod initiation causes heart rate (HR) slowing by 10–15 beats per minute [13, 22, 32, 33]. However, fingolimod initiation is also known to cause bradycardia in 0.5–2.4%, cardiovascular serious adverse events in 0.9%, and atrioventricular blocks in 0.4% of patients [13, 21].

Since cardiovascular autonomic function is frequently compromised in patients suffering from multiple sclerosis (MS) [10, 15, 18, 23, 31], immune-modulating therapies with effects on cardiovascular function might aggravate autonomic dysregulation in MS patients [10]. Particularly orthostatic intolerance and syncope [10] might be triggered by vagomimetic fingolimod effects since reflex syncope typically results from cardiovagal activation and sympathetic withdrawal [39]. As central autonomic modulation and baroreflex function may be compromised in MS patients [10, 18, 40], vagomimetic fingolimod effects might further alter centrally mediated or baroreflex dependent cardioinhibitory responses and thus increase the risk of syncope in MS patients.

However, we previously showed that vagomimetic effects during the first 6 hours upon fingolimod initiation may even yield beneficial changes in the overall autonomic modulation, sympathetic-parasympathetic balance, and baroreflex sensitivity (BRS) under resting conditions [20].

Yet, it is still unknown whether cardiovascular fingolimod effects might compromise autonomic adjustment to situations that require swift, successive increases and decreases of HR and blood pressure (BP), i.e., cardiovascular changes that occur during Valsalva-like maneuvers such as defecation [8], heavy lifting [5, 26], or coughing [36].

Our previous study showed steady HR slowing up to 5 hours after fingolimod initiation despite a drop in continuous BP, i.e., despite baroreceptor unloading [20]. Based on this observation, we hypothesize that direct fingolimod effects on cardiomyocytes and thus on HR might override baroreflex mediated cardiovagal withdrawal upon rapid BP decrease, i.e., that direct myocardial effects of fingolimod may impede rapid HR acceleration during situations associated with a rapid BP decrease. As a consequence of such hypothesized mechanisms, fingolimod might promote syncopal attacks [8, 29, 36]. While we previously observed that fingolimod initiation is associated with a significant increase in spontaneous BRS under resting conditions [20], it is still unclear whether and to which extent fingolimod affects cardiovagal activity during the aforesaid rapid BP challenges, particularly during the first 6 hours after fingolimod initiation.

We therefore revisited the recordings of our previous study before and during the first 6 hours after fingolimod initiation [20] and evaluated whether the cardiovascular fingolimod effects alter the ability to rapidly re-adjust HR and BP in response to sudden baroreflex unloading and subsequent baroreflex loading as it occurs during Valsalva maneuvers (VM).

## Patients and methods

In the group of 21 RRMS patients enrolled in our previous study [20], we analyzed the changes in electrocardiographic RR intervals (RRIs), systolic and diastolic blood pressures during VMs that were performed before and 0.5, 1, 2, 3, 4, 5, and 6 hours after the first dose of fingolimod.

These 21 RRMS patients (14 women and 7 men, mean age 33.5 ± 1.8 years, time since diagnosis 6.0 ± 1.1 years) had been diagnosed with RRMS according to the 2017 revised McDonald criteria and were about to start treatment with fingolimod [20]. They had been selected among the out-patients seen at the Multiple Sclerosis Clinic of the Department of Neurology, University of Erlangen-Nuremberg, Germany. To avoid any misinterpretation of possible fingolimod-related autonomic effects, we had excluded patients with dysautonomia either due to MS itself [15, 20, 31] or to other diseases, such as diabetes or hypertension, or who were on any medication possibly influencing autonomic modulation, such as antidepressants, asthma medications, and cholinesterase inhibitors [20].

We therefore had only included patients in whom cardiovascular autonomic testing, uroflowmetry, assessment of residual urine volume, and the Composite Autonomic Symptom Score 31 (COMPASS 31), an autonomic questionnaire [38], had shown normal results prior to fingolimod initiation [20]. Since there is no standardized cut-off value defining normal results in the COMPASS 31, we excluded patients with clinically manifest autonomic symptoms. Moreover, none of the patients included in this study had any electrocardiographic abnormalities prior to fingolimod initiation [20].

While our previous study [20] had analyzed fingolimod effects on heart rate, BP, sympathetic and parasympathetic cardiovascular modulation and BRS under resting conditions, we now evaluated cardiovascular responses to Valsalva maneuvers, since VM simulates common daily situations that are associated with sudden baroreflex unloading, due to BP decrease upon expiratory strain onset, and with rapidly following baroreflex loading, due to BP overshoot after strain release [37]. We had excluded patients with conditions that are contraindications of performing a VM, such as retinopathy, glaucoma, aneurysms, dissections, or increased intracranial pressure [1, 12, 19]. Moreover, we excluded patients from the study if they were unable to maintain the adequate expiratory pressure during the Valsalva maneuver or if they were not compliant for other reasons. In patients who had been on any previous disease modifying therapy, we discontinued the previous medication for a long enough period to meet current recommendations [28].

The study was approved by the Ethics Committee of the University of Erlangen-Nuremberg, and registered at the German Clinical Trial Register (DRKS00004548) [20]. After a detailed explanation of the study and all its procedures, all study participants gave their written informed consent according to the declaration of Helsinki [20].

### Assessment of MS severity

As previously described [20], we had evaluated the MS severity in all patients by means of the expanded disability status scale (EDSS) [24] and the MS functional composite (MSFC) score [6]. MSFC is composed of three parts: the timed 25-ft walk (7.6 m), the 9-hole peg test, and the paced auditory serial addition test. For each of the three components, we assessed raw scores which were then converted to *z*-scores using the National Multiple Sclerosis Society Task Force MS population as the reference population [11]. Finally, we averaged the three *z*-scores to determine the overall *z*-score of MSFC [11].

#### Cardiovascular recordings

Cardiovascular autonomic testing had been performed under standardized conditions between 9 a.m. and 4 p.m., in a quiet room with an ambient temperature of 24 °C and stable humidity [20]. To assure stable resting conditions, patients had been asked to rest for at least 40 min before we started testing [20]. In all patients, we monitored HR as electrocardiographic RR intervals (RRIs), systolic and diastolic beat-to-beat blood pressure (BPsys, BPdia) by finger-pulse photoplethysmography (Portapress; TPD-Biomedical Instrumentation, Amsterdam, NL), and respiratory frequency (RESP, [min^−1^]) by chest impedance measurements [20] at supine rest and during Valsalva maneuvers (VM). Signals were recorded before and 0.5, 1, 2, 3, 4, 5, and 6 h after fingolimod initiation [20].

As described before [20], bio-signal data were digitalized and displayed on a personal computer and a custom designed data acquisition and analysis system (SUEmpathy™, SUESS-Medizintechnik, Germany) and stored for off-line analysis [18].

#### Valsalva maneuver

As mentioned above, we used a VM to evaluate the rapid, baroreflex-mediated HR acceleration in response to the rapid fall in BP that occurs during the initial phase of strain and to assess baroreflex-mediated HR slowing in response to the BP overshoot occurring upon strain release [9, 19]. The baroreflex-mediated cardiovascular changes during VM are similar to the changes induced by common day-to-day straining activities such as coughing, lifting heavy loads [5, 26], or defecation [8, 9, 36]. Similar to VM, these activities may induce syncope due to the increased intrathoracic pressure with a subsequent decrease in cardiac output, blood pressure, and cerebral blood flow [27, 30], but are not easily standardized and reproduced. In contrast, VM can be standardized, is non-invasive, does not require pharmacological stimulation, and can be repeated within short intervals until reproducible results are obtained [9, 14, 19]. Consequently, our patients performed 2 or 3 VMs at each of the eight time points of recording until at least two VMs showed reproducible results [17]. VM was standardized by asking the participants to blow into a mouthpiece connected to an aneroid manometer and to maintain a pressure of 40 mmHg for 15 s [19]. To assure adequate expiratory pressure, the examiners closely monitored the gauges of the aneroid manometer.

The cardiovascular responses to a VM include four phases. In normal subjects, the mechanically induced sudden rise of intrathoracic pressure results in a brief increase in BP followed by a brief parasympathetically mediated HR decrease (phase 1) [9, 19]. The continued expiratory strain reduces venous cardiac return and thus left ventricular stroke volume and cardiac output. This results in a rapid BP fall (VM phase 2 early) which unloads baroreceptors and activates baroreflex-mediated cardiovagal withdrawal and sympathetic activation resulting in HR acceleration and peripheral vasoconstriction with subsequent BP re-increase (VM phase 2 late) [9, 19]. HR acceleration is mainly due to cardiovagal withdrawal during VM phase 2 early but also results from increased sympathetic output during VM phase 2 late [9, 19, 25]. In healthy individuals, VM phase 2 early ends with the onset of BP recovery which defines the onset of VM phase 2 late. To determine the end of VM phase 2 early and the onset of VM phase 2 late, we therefore identified the nadir of the BP decrease during strain from the raw data of the recordings. The end of expiratory strain causes release of intrathoracic pressure with a subsequent brief fall in BP and a further, baroreflex-mediated HR increase for few beats constituting VM phase 3 [9, 19, 25]. After strain release, during VM phase 4, BP shows a rebound overshoot due to the persistent arteriolar vasoconstriction and the increase in cardiac output that occurs upon release of the expiratory strain [9, 19, 25]. The BP overshoot in VM phase 4 induces baroreceptor loading which triggers cardiovagal activation and sympathetic withdrawal, and thus yields normalization of HR and BP [9, 19, 25].

Since artifacts are inevitable in repeated measurements at eight time points, we only included VM recordings with no or minimal artifacts that would not compromise data analysis, and calculated the following parameters:

- The highest BPsys, BPdia, and RRI values during VM phase 1 (VM1-BPsys_max; VM1-BPdia_max VM1-RRI_max),
- The lowest BPsys, BPdia, and RRI values during VM phase 2 early (VM2early-BPsys_min, VM2early-BPdia_min, VM2early-RRI_min),
- The highest BPsys and BPdia values and the lowest RRI values at the end of phase 2, termed VM phase 2 late (VM2late-BPsys_max; VM2late-BPdia_max, VM2late-RRI_min),
- The highest BPsys, BPdia values due to overshoot in VM phase 4 as well as the highest RRI values (reflecting the slowest heart rate) after strain release in VM phase 4 (VM4-BPsys_max; VM4-BPdia_max, VM4-RRI_max).

As index of baroreflex-mediated vagal withdrawal upon baroreceptor unloading in VM phase 2early, we calculated the baroreflex cardiovagal gain (BRG) by performing a linear regression analysis between the decreasing BPsys values and the decreasing RRI values reflecting the baroreflex-mediated HR increase during VM phase 2 early. We determined BRG as the slope of the correlation between RRI and BPsys values provided the correlation coefficient R^2^ exceeded 0.80 [14, 37].

As index of parasympathetic activation in response to baroreceptor loading by the BPsys overshoot that occurs after strain release, during the initial VM phase 4, we calculated the Valsalva ratio (VR) which is defined as the ratio between the highest HR, i.e., lowest RRI during VM and the lowest HR, i.e., highest RRI within the first 30 s after release of the expiratory strain [19, 25].

### Statistical analysis

Data were tested for normal distribution using the Shapiro-Wilk test. In case of normally distributed data, we assessed differences between corresponding bio-signals or autonomic parameters assessed at the eight time points, i.e., before and 0.5, 1, 2, 3, 4, 5, 6 hours after fingolimod initiation, by means of analyses of variance for repeated measurements (ANOVA, general linear model), and used “assessments” (before, 0.5, 1, 2, 3, 4, 5, and 6 hours after fingolimod initiation) as *within-subject* factor. Suitability of the ANOVA was determined by Mauchly’s test of sphericity. We employed the Greenhouse-Geisser correction in case of violation of the sphericity assumption. In all 21 patients, we performed post hoc paired *t* tests to evaluate differences between values sampled before fingolimod initiation and values sampled at each of the seven time points after fingolimod initiation.

If data were not normally distributed, we used the Friedman test to assess differences between values sampled at the time points before and after fingolimod initiation, and then used Wilcoxon tests to evaluate differences between values recorded before fingolimod initiation and values sampled at each of the seven time points after fingolimod initiation.

## Results

As mentioned in our previous study [20], all the 21 RRMS patients completed bio-signal recordings before and after 0.5, 1, 2, 3, 4, 5, and 6 hours of fingolimod initiation. Their median EDSS score was 2.0 with an interquartile range (IQR) of 1.5–3. The MSFC z-scores were 0.16 ± 0.09 [20]. No patient developed symptomatic bradycardia, arrhythmia, pre-syncopal or syncopal symptoms during the first 6 hours after fingolimod initiation [20].

### Bio-signals during Valsalva maneuver, baroreflex gain, and Valsalva ratios assessed before and within 6 hours after fingolimod initiation

#### VM phase 1

Already 30 min after fingolimod initiation, maximal RRIs during VM phase 1 (VM1-RRI_max) were significantly higher than VM1-RRI_max before fingolimod initiation (902.2 ± 181.9 vs. 814.53 ± 124.69 ms; *p* < 0.05; Table 1). VM1-RRI_max further increased after 1, 2, and 3 hours and reached peak values after 4 hours, then started to decline but was still higher after 5 and 6 hours than pre-fingolimod VM1-RRI_max values (Table 1).

**Table 1:** RR intervals (RRIs), systolic and diastolic blood pressures (BPsys, BPdia), at phases 1, 2 early, 2 late, and 4 of Valsalva maneuvers (VM), as well as Valsalva ratios and baroreflex gain at VM phase 2 early performed at eight time points before and after fingolimod initiation

Parameters	Time after fingolimod initiation (hour)								
	Before	0.5	1	2	3	4	5	6	*p* values
VM phase 1									
Max RRI (ms)	814.5 ± 124.6	900.59 ± 44.60*	947.0 ± 157.5**	930.0 ± 112.6**	989.9 ± 132.1***	1020.9 ± 150.1***	1004.9 ± 159.1***	997.8 ± 204.5**	0.000 (A)
Max BPsys (mmHg)	142.2 ± 12.8	142.94 ± 4.88	139.6 ± 14.2	136.1 ± 13.	131.7 ± 14.2**	128.4 ± 10.3**	131.4 ± 13.3**	132.9 ± 9.8*	0.019 (A)
Max BPdia (mmHg)	81.2 ± 9.5	80.62 ± 3.05	72.6 ± 17.3	74.8 ± 10.6*	70.1 ± 12.6**	61.8 ± 17.8***	66.7 ± 10.2**	68.8 ± 6.9**	0.000 (A)
VM phase 2 early									
Min RRI	638.6 ± 81.3	725.18 ± 113.32***	758.3 ± 106.1***	738.6 ± 122.9***	772.4 ± 94.9***	780.8 ± 79.4***	752.3 ± 138.8***	775.1 ± 131.2***	0.000 (A)
Min BPsys (mmHg)	98.5 ± 16.5	95.53 ± 5.37	96.0 ± 21.9	95.3 ± 15.4	91.9 ± 19.7	89.8 ± 17.3	88.2 ± 20.4	93.3 ± 16.9	0.422 (A)
Min BPdia (mmHg)	61.9 ± 8.0	60.30 ± 3.04	59.5 ± 11.7	56.8 ± 7.9	51.4 ± 9.9***	47.9 ± 8.0**	46.8 ± 9.4***	50.9 ± 5.2**	0.001 (A)
VM phase 2 late									
Min RRI (ms)	584.1 ± 84.5	618.63 ± 25.08**	658.9 ± 108.3***	671.1 ± 126.1**	694.6 ± 106.8***	698.8 ± 121.6**	691.0 ± 133.0**	695.8 ± 149.6**	0.028 (A)
Max BPsys (mmHg)	116.0 ± 25.6	111.22 ± 6.73	114.3 ± 22.4	115.5 ± 20.0	105.3 ± 20.6*	104.7 ± 19.7	101.7 ± 21.9*	107.2 ± 19.9	0.216 (A)
Max BPdia (mmHg)	75.2 ± 13.0	72.80 ± 4.11	72.1 ± 13.6	68.1 ± 10.4	62.3 ± 11.5***	59.2 ± 7.0***	57.6 ± 9.4***	62.9 ± 7.8**	0.001 (A)
VM phase 4									
Max RRI (ms) after strain release	992.7 ± 150.7	936.53 ± 57.77	989.1 ± 176.9	1028.9 ± 147.3	1012.2 ± 110.0	1021.6 ± 146.2	999.7 ± 140.2	998.0 ± 147.0	0.850 (F)
Max BPsys overshoot (mmHg)	147.2 ± 18.0	147.78 ± 5,87	144.3 ± 21.7	143.8 ± 15.0	137.1 ± 15.9**	139.8 ± 18.0	131.5 ± 14.9**	138.2 ± 13.0**	0.015 (A)
Max BPdia overshoot (mmHg)	80.6 ± 9.1	82.47 ± 3.20	82.0 ± 12.2	77.9 ± 9.7	71.2 ± 9.3***	69.2 ± 13.0**	69.5 ± 10.9**	72.9 ± 8.7**	0.002 (A)
Valsalva ratios	1.78 ± 0.4	1.66 ± 0.14	1.57 ± 0.6	1.68 ± 0.5	1.63 ± 0.4*	1.57 ± 0.4**	1.56 ± 0.4**	1.57 ± 0.4**	< 0.001(F)
BRG (ms/mmHg) in VM phase 2 early	3.9 ± 0.5	3.8 ± 0.6	3.9 ± 0.5	4.7 ± 0.4	4.8 ± 0.44^#^	5.4 ± 0.77*	5.1 ± 0.6^#^	3.9 ± 0.6	0.285 (A)

RR intervals (RRIs), systolic and diastolic blood pressures (BPsys, BPdia), at phases 1, 2 early, 2 late, and 4 of Valsalva maneuvers (VM) performed at eight time points before and after fingolimod initiation. Valsalva ratios were calculated as ratios between the lowest RRI, i.e., highest heart rate, at the end of expiratory strain and the highest RRI, i.e., lowest heart rate, after strain release. Baroreflex gain (BRG) was calculated by linear regression analysis between decreasing BPsys values and decreasing RRI values during VM phase 2 early, reflecting the baroreflex-mediated HR increase upon BP decrease

Data were expressed as mean ± standard deviation (SD). *p* values in the right column relate to the results of ANOVA (A) or Friedman (F) tests. The number of asterisks indicates the level of significance of post hoc analyses, with * indicating *p* < 0.05, ** p < 0.01, and *** p < 0.001

ANOVA showed no significant difference between BRGs at the eight time points (*p* = 0.285). Only exploratory *t* tests between BRG values before and after fingolimod initiation showed higher slightly BRGs after 3 hours (*p* = 0.064) and 5 hours (*p* = 0.064) and significantly higher BRG after 4 hours (*p* = 0.027)

Maximal BPsys and BPdia values during VM phase 1 (VM1-BPsys_max and VM1-BPdia_max) were slightly lower 1 hour after fingolimod initiation than before fingolimod initiation. The decrease was significant after 3, 4, 5, and 6 hours for VM1-BPsys_max, and after 2, 3, 4, 5, and 6 hours forVM1-BPdia_max and most prominent after 4 hours for both parameters.

#### VM phase 2 early

Already 30 min after fingolimod initiation, the lowest RRI values during VM phase 2 early (VM2early-RRI_min) were significantly higher than the respective value before fingolimod initiation (638.6 ± 81.3 ms). VM2early-RRI_min values further increased 1 to 6 hours after fingolimod initiation and were highest after 4 hours (780.82 ± 79.41 ms; *p* < 0.001; Table 1).

The lowest BPsys values during the VM phase 2 early, (VM2early-BPsys_min) were not significantly lower during hours 1 to 6 after fingolimod initiation than before fingolimod initiation. In contrast, the lowest BPdia values during the VM phase 2 early, (VM2early-BPdia_min) were significantly lower 3, 4, 5, and 6 hours after than before fingolimod initiation (61.9 ± 7.9 mmHg) and showed the lowest values after 5 hours (46.8 ± 9.4 mmHg).

#### VM phase 2 late

The lowest RRI values at the end of VM phase 2 (VM2late-RRI_min) were higher at all time points after than before fingolimod initiation (584.1 ± 84.5 ms; *p* < 0.01). The difference was most pronounced after 4 hours (698.8 ± 121.6 ms).

ANOVA showed no significant difference between the highest BPsys values at the end of VM phase 2 (VM2late-BPsys_max) at the eight time points before and after fingolimod initiation. However, exploratory *t* tests showed significantly lower values of VM2late-BPsys_max 3 and 5 hours after than before fingolimod initiation (116.0 ± 25.6 mmHg). The highest BPdia values at the end of VM phase 2 (VM2late-BPdia_max) were significantly lower 3, 4, 5, and 6 hours after than before fingolimod initiation (75.2 ± 13.0 mmHg). The difference was most prominent after 5 hours (57.6 ± 9.0; *p* < 0.001; Table 1; Fig. 3).

#### VM phase 4

The highest RRI values during VM phase 4, (VM4-RRI_max, reflecting the lowest HR after strain release), were not significantly higher after than before fingolimod initiation (Table 1; Fig. 4).

In contrast, the highest BPsys values during VM phase 4 (VM4-BPsys_max reflecting peak B*P* values during overshoot) were significantly lower 3, 5, and 6 hours after fingolimod initiation than VM4-BPsys_max before fingolimod initiation (147.2 ± 18.0 mmHg). VM4-BPsys_max was lowest after 5 hours (131.5 ± 14.9 mmHg). The highest BPdia values upon strain release, (VM4-BPsys_max) showed a more prominent lowering after fingolimod initiation: VM4-BPdia_max values were significantly lower 3, 4, 5, and 6 hours after than before fingolimod initiation. The difference to pre-fingolimod VM4-BPdia_max (80.6 ± 9.0 mmHg) was most pronounced after 4 hours (VM4-BPdia_max: 69.2 ± 13.0 mmHg; Table 1; Fig. 4).

#### Valsalva ratio

Already 30 min after fingolimod initiation, the Valsalva ratio (VR) was slightly but insignificantly lower than VR before fingolimod initiation (1.8 ± 0.4). After 3, 4, 5, and 6 hours, VR was significantly lower than pre-fingolimod VR (Table 1; Fig. 5).

#### Baroreflex gain during VM phase 2 early

ANOVA showed no significant difference between BRGs at the eight time points (*p* = 0.285). Only exploratory *t* tests between BRG values before and after fingolimod initiation showed slightly higher BRGs after 3 hours (4.81 ± 0.44; *p* = 0.064) and 5 hours (5.12 ± 0.6; p = 0.064), and significantly higher BRG after 4 hours (*p* = 0.027) compared to the BRG before fingolimod initiation (3.9 ± 2.0 ms/mmHg; Table 1; Fig. 5).

## Discussion

Our study confirms cardiovascular effects of fingolimod on the maximal and minimal RRI, BPsys and BPdia values during VM. Compared to values before fingolimod initiation, RRIs after fingolimod initiation were higher, i.e., HR values were lower, during all VM phases except for the longest RRIs, i.e., slowest HR, after strain release during VM phase 4.

**Fig. 1:**
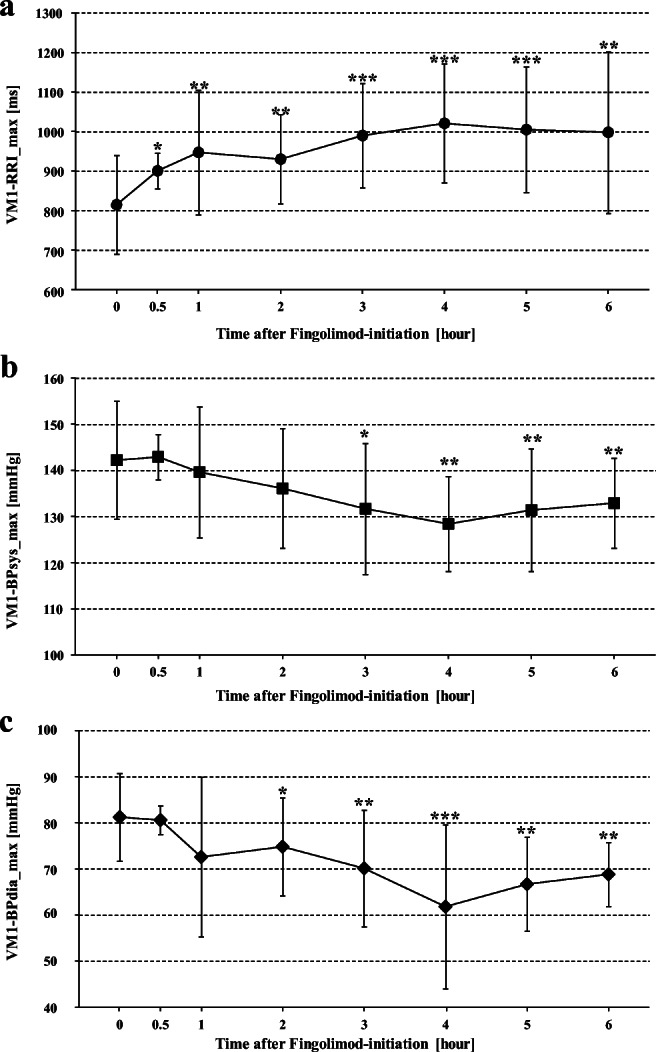
Maximum RR intervals (RRIs), systolic and diastolic blood pressures (BPsys, BPdia), at phase 1 of Valsalva maneuvers (VM) performed at eight time points before and after fingolimod initiation. The number of asterisks indicates the level of significance of post hoc analyses, with * indicating *p* < 0.05, ** p < 0.01, and *** p < 0.001

**Fig. 2:**
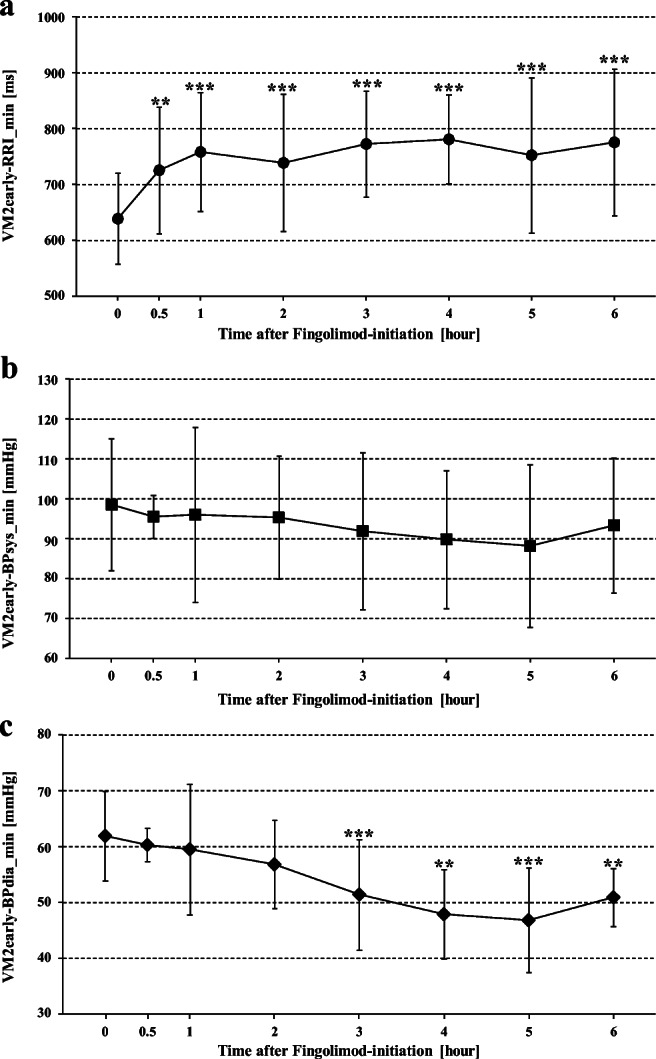
Minimum RR intervals (RRIs), systolic and diastolic blood pressures (BPsys, BPdia), at phase 2 early of Valsalva maneuvers (VM) performed at eight time points before and after fingolimod initiation. The number of asterisks indicates the level of significance of post hoc analyses, with * indicating *p* < 0.05, ** p < 0.01, and *** p < 0.001

**Fig. 3:**
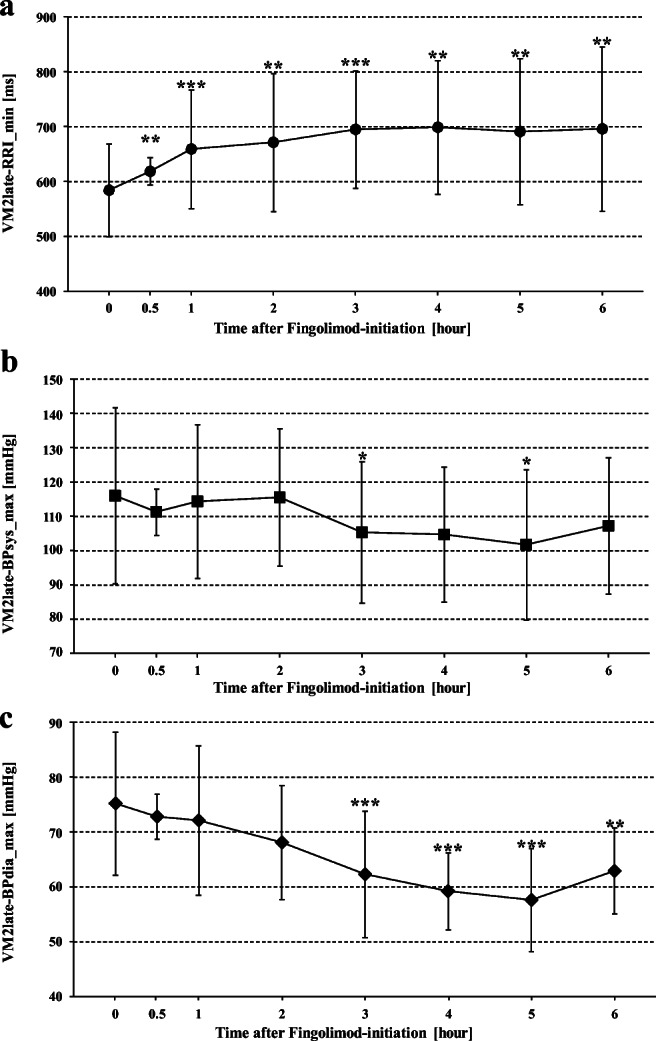
Minimum RR intervals (RRIs), maximum systolic and diastolic blood pressures (BPsys, BPdia), at phase 2 late of Valsalva maneuvers (VM) performed at eight time points before and after fingolimod initiation. The number of asterisks indicates the level of significance of post hoc analyses, with * indicating *p* < 0.05, ** p < 0.01, and *** p < 0.001

**Fig. 4:**
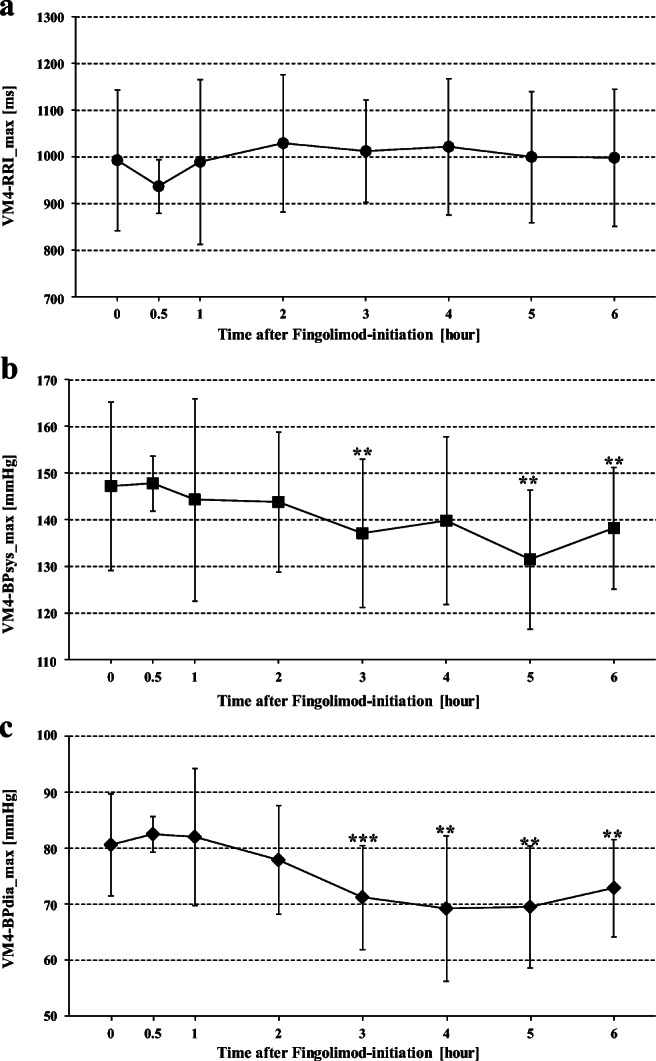
Maximum RR intervals (RRIs), systolic and diastolic blood pressures (BPsys, BPdia), at phase 4 of Valsalva maneuvers (VM) performed at eight time points before and after fingolimod initiation. The number of asterisks indicates the level of significance of post hoc analyses, with * indicating *p* < 0.05, ** p < 0.01, and *** p < 0.001

**Fig. 5:**
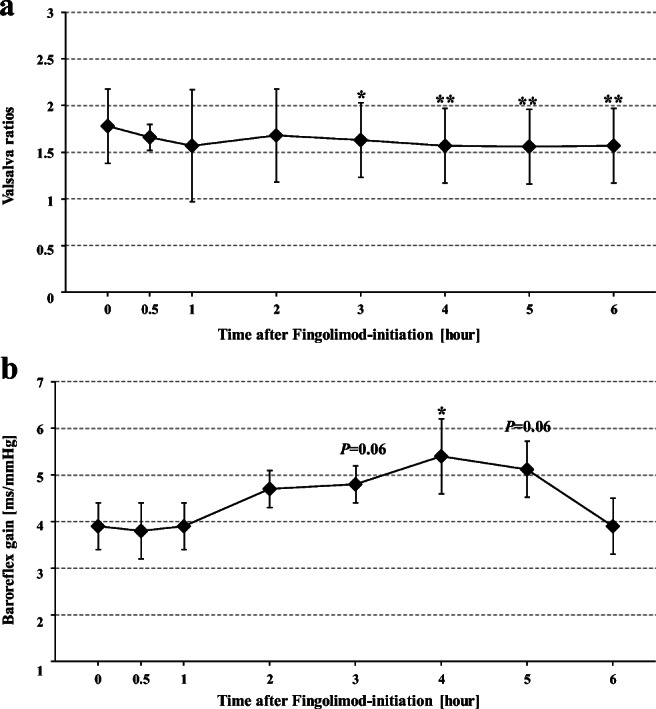
Valsalva ratios and baroreflex gain at phase 2 early of Valsalva maneuvers (VM) performed at eight time points before and after fingolimod initiation. The number of asterisks indicates the level of significance of post hoc analyses, with * indicating *p* < 0.05, ** p < 0.01, and *** p < 0.001

After 3 to 6 hours, particularly BPdia values of VM phase 1, VM phase 2 early, VM phase 2 late, and the BP overshoot during VM phase 4 were also lower than corresponding values before fingolimod initiation (Table 1; Figs. 1, 2, and 3). BPsys values decreased less but still were lower at the end of strain (VM phase 2 late) and during overshoot (VM phase 4) 3, 5, and 6 h after fingolimod initiation than corresponding values before fingolimod initiation (Table 1; Figs. 3 and 4). However, BRG remained stable during most time points after fingolimod initiation and was even significantly higher after 4 hours than BRG before fingolimod initiation (Table 1; Fig. 5). In contrast, VR was significantly lower 4 to 6 hours after than before fingolimod initiation (Table 1; Fig. 5).

To some extent the fingolimod-related changes in RRI and BP responses to VM are similar to the fingolimod-induced cardiovascular and autonomic changes that we previously found in the same patient group under supine resting conditions [20]. During the first 6 hours after fingolimod initiation, we had seen the well-known slowing of heart rate, i.e., increase in RRIs, no significant changes in BPsys but a significant decrease in diastolic BP particularly 3 to 6 hours after fingolimod initiation [20]. However, parasympathetic and overall cardiac autonomic modulation as well as spontaneous baroreflex sensitivity had even increased 1 to 6 hours after fingolimod initiation. We had therefore concluded that autonomic changes upon fingolimod initiation might even have beneficial effects on the cardiovascular system under resting conditions [20]. Thus, the relative RRI and BP changes during the VMs at the eight time points resemble the changes of RRIs and BP recorded at the eight time points before and after fingolimod initiation under resting conditions [20].

### Fingolimod decreases Valsalva ratios

These changes explain why VR started to decrease already 30 min after fingolimod initiation and was significantly lower than pre-fingolimod VR (1.8 ± 0.4) when fingolimod had its most prominent effects on HR and BP [2, 10, 13, 20], i.e., 3 to 6 hours after fingolimod initiation (Table 1; Figs. 1, 2, 3, and 5). Similar to the fingolimod effects on RRIs, BPsys and BPdia under resting conditions [20], vagomimetic fingolimod effects prolonged RRIs, i.e., slowed HRs, at VM phases 1, 2 early, and 2 late (Table 1) most prominently after 3 to 5 hours [2, 10, 13, 20]. Fingolimod-induced HR slowing also affected maximum BPsys at VM phase 1, VM phase 2 late, and during VM phase 4, i.e., during BP overshoot although BPsys decreases from pre-fingolimod values were not significant at all time points (Table 1; Figs. 1, 3, and 4). The slight decrease in BPsys values during VM can be explained by the decrease in cardiac output associated with the fingolimod-induced HR slowing [16]. In contrast, fingolimod had more prominent effects on BPdia since fingolimod also activates endothelial S1P1 receptors and thus enhances release of endothelium-dependent nitric oxide which mediates arterial vasodilation [2, 20]. The resulting decrease in peripheral resistance significantly lowered BPdia [2, 20] in VM phase 1, VM phase 2 early, VM phase 2 late, and during the VM phase 4 overshoot (Table 1) already 2 or 3 hours after fingolimod initiation and induced a further BPdia decrease after 4, 5, and 6 hours upon fingolimod initiation (Table 1; Figs. 1, 2, 3, and 4).

In contrast to all RRI values during expiratory strain, the highest RRI values, i.e., slowest HRs, after strain release did not change significantly after fingolimod initiation but were similar at all time points before and after fingolimod initiation (Table 1; Fig. 4). Consequently, VR, i.e., the ratio between the highest and lowest RRI values had to be smaller after than before fingolimod initiation: since vagomimetic fingolimod effects on the lowest RRIs (i.e., the highest HR) at the end of strain (VM2late-RRI_min) were most prominent 3 to 5 hours after fingolimod initiation [2, 10, 13, 20], the ratio between the lowest RRI (i.e., highest HR) at the end of strain and the—unchanged—highest RRI (i.e., the lowest HR) after strain release, i.e., on VM4-RRI_max, had to be significantly smaller 3–6 hours after fingolimod initiation compared to the Valsalva ratio before fingolimod initiation (Table 1; Fig. 5).

Usually, a decrease in VR indicates autonomic dysfunction with impaired cardiovagal buffering of baroreceptor loading by increased BP [9, 17].

However, the decreased VR after fingolimod initiation does not imply compromised baroreflex function. Instead, the decrease in VR after fingolimod initiation is not necessarily related to insufficient parasympathetic activation in response to the BP overshoot after strain release but can be explained by the fingolimod-induced attenuation of HR increase during VM phase 2 late. Evidently, vagomimetic fingolimod effects on HR and vasodilating effects on BPdia partially buffered autonomic responses to baroreceptor unloading during VM phase 2 early [2, 10, 13, 20] and yielded lower HR (i.e., higher RRI values) and BPs at the end of strain (Table 1) after than before fingolimod initiation. Less BP overshoot, at the end of strain (i.e., lower BP at VM phase 4) requires less baroreflex-mediated HR slowing after strain release [9, 17].

After fingolimod initiation, the lowest HRs during VM phase 4, i.e., the maximum RRIs after strain release, therefore, did not differ significantly from corresponding pre-fingolimod values (Table 1, Fig. 4). Consequently, the decreased Valsalva ratios 3 to 6 hours after fingolimod initiation can be interpreted as a physiologic response to the attenuated BP overshoot in VM phase 4 after fingolimod initiation. Moreover, the Valsalva ratio always remained within the normal age-related ranges for men and women [25], and none of our patients experienced any clinical cardiovascular or pre-syncopal symptoms during or after the VMs performed under the influence of fingolimod.

Moreover, the baroreflex gain assessed by calculating the regression between the decreasing BP values and the decreasing RRI values, i.e., the increasing HR values, during VM phase 2early, did not deteriorate after fingolimod initiation. Instead, BRG remained stable and was slightly higher 3 hours (4.81 ± 0.44; *p* = 0.064), significantly higher 4 hours (5.39 ± 0.77; *p* = 0.027), and again slightly higher 5 hours (5.12 ± 0.6; p = 0.064), after fingolimod initiation than before fingolimod initiation (3.92 ± 0.5; Fig. 5).

Two physiologic changes might contribute to the stable or increased in BRG during VM phase 2 early, i.e., during the sudden, strain-induced drop in BP.

First, we previously found a significant increase in resting baroreflex sensitivity already 1 hour and even more 2 to 6 hours after fingolimod initiation [20]. BRS at rest was highest when fingolimod had shifted HR and BP values to their nadir [20]. We concluded that the increase in spontaneous baroreflex sensitivity might result from central adjustment of efferent baroreflex responses to the steadily increasing vagomimetic fingolimod effects [4, 20].

The fingolimod-induced steady decrease of resting HR and BPs 2, 3, and 4 hours after fingolimod initiation [20] implies a shift of HR and BP values on the sigmoid baroreflex curve. This shift evidently moved the reflex operating point closer towards the point of maximal gain [9], and resulted in the highest BRS when HR and BP were at their nadir [20, 34].

Second, and in addition to the resetting of spontaneous BRS at rest, i.e., prior to VM, the swift BP decrease during VM phase 2 early may also influence baroreflex gain [3, 35]. In dogs, Chapleau and Abboud demonstrated that BP oscillations shift the operating threshold of the baroreflex to BP values below the operating threshold of static BP [3]. The authors also observed higher firing rates in canine sinus nerves when low pulsatile instead of low static BP was applied to the baroreceptors [3]. While BP changes during VM are not pulsatile, the rather swift change from a brief BP increase in VM phase 1 to the subsequent BP decrease in VM phase 2 early might have some effect on the baroreflex sensitivity similar to the findings of Chapleau and Abboud [3]. In addition, the rapid baroreflex unloading from increased BP during the brief VM phase 1 to decreased BP at the end of VM phase 2 early might add to BRS resetting, similar to the resetting reported upon orthostasis [35]. Upon head-up tilt, Schwartz and Stewart did not see a prominent increase in cardiovagal baroreflex sensitivity but only an insignificant increase in the cardiovagal slope, i.e., the heart rate change per BP change due to baroreceptor unloading [35]. However, the authors recorded a significant increase in muscle sympathetic nerve activity bursts per mmHg change in BP compared to the sympathetic responses to spontaneous BP changes in supine position [35]. This change in sympathetic baroreflex sensitivity upon rapid baroreceptor unloading might explain why we found an increase in BRG during VM phase 2 early despite the concurrent vagomimetic effects of fingolimod that counteracted to some extent the effects of parasympathetic withdrawal on HR acceleration. Although HR at the end of VM phase 2 late had not accelerated to values as high as HR at the end of VM phase 2 late before fingolimod initiation, BRG was still significantly higher after 4 hours, i.e., when vagomimetic effects were most pronounced and HR at its nadir, than BRG before fingolimod initiation.

We assume that this BRG increase was predominantly owed to an increase in sympathetic baroreflex sensitivity, similar to the increase observed during head-up tilt [35]. Chapleau et al. suggest that the change in BRS is largely due to a change in central command, i.e., a central adjustment of baroreflex output [4].

## Conclusion

In summary, fingolimod does not compromise baroreflex-mediated rapid cardiovascular adjustment to baroreceptor unloading and subsequent baroreceptor loading. Fingolimod has significant effects on HR and BP not only under resting conditions [20] but also during all phases of VM. However, improved spontaneous BRS at resting conditions [20] and stable or increased BRG at times when HR and BP values of VM were at their nadir resulted in adequate cardiovascular recovery and prevented presyncope or syncope in our MS patients.

### Limitations of our study

Still, we cannot rule out that fingolimod might yield clinically relevant pre-syncopal or syncopal effects during longer lasting or more pronounced baroreceptor unloading, for example during prolonged VMs, or VM-like activities associated with more severe expiratory straining, such as heavy lifting, coughing, or abdominal straining during defecation [5, 8, 9, 26, 36]. Then, vagomimetic fingolimod effects might theoretically impede adequate HR acceleration while vasodilating fingolimod effects [2] might delay sympathetic vasoconstriction. Subsequently, HR and BP recovery from baroreflex unloading might be inadequate or critically prolonged.

Expiratory strain increases intrathoracic pressure which results in reduced venous return, lowered cardiac output and BP which, in turn, decreases blood flow to the brain [30, 37]. In healthy persons, the strain of a VM performed in supine position with 40 mmHg expiratory pressure—as used in our patients—reduces the middle cerebral artery blood flow velocity by approximately 35% [30]. Pott et al. demonstrated that rather mild and common additional challenge such as standing, i.e., additional baroreceptor unloading, further decreases middle cerebral artery blood flow during strain to approximately 50% of values at supine rest [30]. Since MS patients often have autonomic dysfunction [10, 15, 18, 23, 31], one might assume that fingolimod effects on BP recovery and HR increase during VM phase 2 late could add to the risk of syncope. However, our patients had rather mild MS stages with a median EDSS score of 2.0 (IQR 1.5–3) and MSFC z-scores of 0.16 ± 0.09. We cannot rule out an increased risk of syncope upon increased expiratory strain and challenge [7] in patients with more severe MS stages. In our study we did not monitor changes in cardiac output or cerebral blood flow velocities during VM nor did we compare cardiovascular parameters upon fingolimod initiation between MS patients and controls, which would be difficult for ethical reasons. Still, the findings of improved spontaneous BRS at rest [20], of stable or even increased BRG upon baroreflex unloading, and the finding that VR remained within normal limits during the first 6 hours after fingolimod initiation support the conclusion that expiratory strain of 40 mmHg does not expose MS patients who start taking fingolimod to an increased risk of syncope.
